# Antimicrobial activity of different Finnish monofloral honeys against human pathogenic bacteria

**DOI:** 10.1111/apm.12039

**Published:** 2012-12-27

**Authors:** Sanna Huttunen, Kaisu Riihinen, Jussi Kauhanen, Carina Tikkanen-Kaukanen

**Affiliations:** 1 Institute of Public Health and Clinical Nutrition, University of Eastern FinlandKuopio, Finland; 2 Ruralia Institute, University of HelsinkiMikkeli, Finland

**Keywords:** Honey, antimicrobial, Streptococcus pneumoniae, Streptococcus pyogenes, Staphylococcus aureus, methicillin-resistant *Staphylococcus aureus*

## Abstract

The antimicrobial activity and phenolic compounds of five Finnish honey products against important human pathogens *Streptococcus pneumoniae*, *S. pyogenes*, *Staphylococcus aureus,* and methicillin-resistant *S. aureus* were analyzed. Microbroth dilution method and HPLC-DAD were used in antimicrobial testing and phenolic compound determination, respectively. Significant antimicrobial activity (p < 0.01) against all the tested pathogens was found from willow herb (*Epilobium angustifolium*), heather (*Calluna vulgaris*), and buckwheat (*Fagopyrum esculentum*) honeys. This is the first report on antimicrobial activity of Finnish monofloral honeys against streptococcal and staphylococcal bacteria. To our knowledge this is also the first report on the antimicrobial effect of honey against *S. pneumoniae*.

*Streptococcus pneumoniae*, an important human pathogen, is the most common cause for pneumonia, meningitis, and otitis media 1. Pneumococcal infections cause death for more than 1 million children in the world per year, especially in developing countries 2. *S. pyogenes*, the group A streptococcus, is a major human pathogen that causes many common, as well as life-threatening illnesses, such as pharyngitis and sepsis, respectively 3. *Staphylococcus aureus* is a common community- and hospital acquired human pathogen that can cause different illnesses from skin infections to severe conditions. Due to its resistance, treatment of infections caused by methicillin-resistant *S. aureus* (MRSA) is extremely difficult 4. Colonization of the nasopharyngeal niche is a commonality shared among *S. pneumoniae*, *S. pyogenes,* and *S. aureus* 5.

Antibiotic resistance is a serious problem worldwide, and it has made the search for new antimicrobial compounds more important 6. Honey has been used as a traditional medicine for centuries 7. Many *in vitro* studies have revealed antimicrobial activity of different honeys against a wide range of skin colonizing and food-borne bacterial species, including antibiotic-resistant bacteria 8–12. Honey has beneficial actions against wound infections also *in vivo* 13, and licensed honey products are widely used in wound care 14.

Several properties in honey contribute to its antimicrobial activity. High osmolarity, low pH, and hydrogen peroxide are the main antimicrobial factors 15,16. Also phenolic compounds may contribute to antimicrobial activity 18. The two medicinal honeys mostly used in wound management, Revamil® and Manuka honeys 19, have additional antimicrobial mechanisms. The main active component in Manuka honey is methylglyoxal 20 and an antimicrobial peptide, bee defensin-1, has been identified from Revamil® honey 12.

Many studies on antimicrobial activity of honey have been conducted in non-European countries 21, and especially in southern hemisphere 9–23. New Zealand Manuka honey is widely studied and used clinically. However, it has been found that other honeys with different floral backgrounds exhibit equivalent inhibitory activity 9. It is thus reasonable to search for new antimicrobial honey candidates from different parts of the world.

In this study, we tested the antimicrobial activity from different Finnish monofloral honeys against important human pathogens: *S. pneumoniae*, *S. pyogenes*, *S. aureus,* and MRSA. The tested honeys were buckwheat honey (*Fagopyrum esculentum*), cloudberry honey (*Rubus chamaemorus*), heather honey (*Calluna vulgaris*), lingonberry honey (*Vaccinium vitis-idaea*), and willow herb honey (*Epilobium angustifolium*). Phenolic compounds in honeys were analyzed by HPLC-DAD method.

## Materials and Methods

### Bacterial strains and culture conditions

*Streptococcus pyogenes* (ATCC 8184), *S. aureus* (ATCC 25923), and MRSA (ATCC 43300) were from ATCC. *S. pneumoniae* clinical strain SB 53845 (isolated from lung) was received from Sauli Haataja (University of Turku, Finland). *S. pyogenes*, *S. aureus*, and MRSA were cultured at 37 °C on sheep blood agar plates (Becton Dickinson, Franklin Lakes, NJ, USA). *S. pneumoniae* was cultured at 37 °C in CO_2_ atmosphere on sheep blood agar plates (Labema Inc., Kerava, Finland) as previously described 24.

### Preparation of the honey samples

Monofloral honey samples were obtained from Finnish Beekeepers Association. Heather (*C. vulgaris*) and lingonberry honeys (*V. vitis-idaea*) were harvested from Western Finland at 63° N (Kaustinen and Pihtipudas, respectively), buckwheat honey (*F. esculentum*) was collected from Eastern Finland at 62° N (Kitee), willow herb honey (*E. angustifolium*) was obtained from Northern Finland at 65° N (Oulu), and cloudberry honey (*R. chamaemorus*) was collected at 68° N (Sodankylä) during the year 2008. Identification of the floral source of honey was based on organoleptic characteristics of the honey and it was supported by pollen analysis. Honey samples were diluted in phosphate buffered saline (PBS) (Gibco, Paisley, UK) containing Todd-Hewitt broth supplemented with yeast extract (THY) (Becton Dickinson, Le Pont de Claix, France). The concentration of THY in PBS was 0.05%. The concentrations of the studied honeys are expressed as the percentage of honey weight per total reaction volume used in the antimicrobial assay (w/v). The tested honey concentrations were 60%, 40%, and 20% (w/v).

### Phenolic standards

The phenolic compounds: *p*-hydroxybenzoic acid (H5376), vanillic acid (V2250), gallic acid (G8647), caffeic acid (C0625), ferulic acid (F3500), *p*-coumaric acid (C9008), 3,4-hydroxybenzoic acid (P5630), (+)-catechin (C1251), (−)-epicatechin (E1753) were purchased from Sigma Chemical Co. (St Louis, MO, USA) and quercetin 3-glucoside (1327S) from Extrasynthese (Genay Cedex, France).

### Microbroth dilution assay

A modification of a microtiter broth microdilution assay described by Amsterdam 2005 25 was chosen for testing the antibacterial activity of different honey samples. The antimicrobial assay was performed as described before 24. Shortly: bacteria were cultured overnight on sheep agar plates and one colony of each bacterial strain was collected into 5 mL of THY broth (*S. pneumoniae* and *S. pyogenes*) or brain heart infusion (BHI) broth (Oxoid, Cambridge, UK) (*S. aureus* and MRSA) for subculture. Then the culture was carried out at 37 °C for 3–4 h or overnight depending on the growth of the bacterial strain. The cultures were followed by measuring A_600_ absorbance values. Each culture inoculum was individually standardized. The densities of the bacterial suspensions were adjusted to the A_600_ values of 0.4–0.7 to achieve colony-forming units (CFUs)/mL approximately of 10^9^–10^13^ depending on the bacterial strain. Then appropriate dilutions were made in PBS to bring the inoculum density to the range of 10^3^–10^4^ CFU/mL depending on the bacterial strain. The diluted bacteria were then inoculated to the test and control samples. Because of the differences in growth between the bacterial pathogens, inhibition induced by honey samples was always compared to the respective bacterial control prepared as the samples, but without honey addition. The controls were made each time for the studied strain in question and carried in the experiment at the same time with the sample. Different concentrations of the honey samples (80 μL) were then mixed with the diluted bacteria (20 μL) and the bacteria-honey mixtures were incubated in microtiter plates (Falcon Flexible Plate; Becton Dickinson Labware) at 37 °C for 2 h. The controls were prepared without adding honey to the reaction mixture or by adding ampicillin as described before 24–26. Shortly: a quantity of 80 μL of 0.05% THY or BHI broth with yeast extract in PBS was mixed with the diluted bacterial suspension of 20 μL (bacteria-broth/PBS control) and incubated in microtiter plate wells as described for the bacteria-honey samples. The ampicillin control was prepared as the bacteria-broth/PBS controls with ampicillin addition to the final concentration of 80 μg/mL. To measure the antibacterial activity of the honeys, all the bacteria-honey samples and the bacteria-broth/PBS controls were plated on sheep blood agar plates in triplicate and the numbers of the colonies were counted next day. Bacterial survival was calculated by comparing the CFU numbers of the bacteria-honey samples with CFU numbers of the bacteria-broth/PBS controls of the respective bacterial strain. Two independent antimicrobial assays were done for each of the studied bacterial strain.

### Extraction of polyphenols

The honey samples (5 g) were weighed in centrifuge tubes and deionized water was added to equilibrate the content of soluble solids to 20 g/100 g in all honey samples. Rare flavonol, morin (1 mg/mL methanol, 0.1 mL) was added as an internal standard, to follow extractability of polyphenols. Extractions were performed with ethyl acetate (4 × 10 mL) by shaking the samples vigorously several times followed by centrifugation. The whole 40-mL portion of the ethyl acetate extract was evaporated to dryness with a rotary evaporator, dissolved in 1 mL of methanol and analyzed with HPLC-DAD.

### HPLC-analysis of honey samples

All samples were filtered through a 0.45-μm syringe filter before injection into the HPLC. The HPLC-DAD apparatus consisted of a Hewlett-Packard instrument with a 1100 series quaternary pump, an autosampler, and a diode array detector linked to an HP-ChemStation data handling system (Waldbronn Analytical Division, Waldbronn, Germany). HPLC separation was achieved on a (150 × 4.6 mm i.d., 5 μm) Phenomenex Gemini reversed-phase (RP) column (RP-18; Merck, Darmstadt, Germany) protected with a guard column of the same material (4 × 3 mm). The linear gradient was based on increase of the organic phase from 5% to 30% during 25 min (acetonitrile and methanol, ACN: MeOH, 85:15, v/v) in the 1% formic acid water phase. Separation was followed by rising the organic phase to 90% during 10 min, returning to initial conditions during 5 min and then by re-equilibration of the column for 10 min. The flow rate was 1.0 mL/min.

HPLC combined with diode array detection was used for UV–Vis spectral analysis and quantification. Identification of the polyphenols in the chromatograms was based on retention times and comparison of their UV–Vis spectral shape, and wavelengths of maximum absorption, and wavelengths of shoulders (sh) of representative standards. The representative standards were analyzed near their wavelengths of maximum absorption: benzoic acid and hydroxybenzoic acids (vanillic acid, *p*-hydroxybenzoic acid, gallic acid, and 3,4-hydroxybenzoic acid) at 260 nm, hydroxycinnamic acids (caffeic acid, ferulic acid, and *p*-coumaric acid,) at 320 nm, flavonol glycosides of flavonoids (quercetin 3-*O*-glucoside) at 360 nm. In honey samples typical spectra neither of ellagic acid, anthocyanins nor flavan-3-ols were detected 27–28. Identified individual compounds were quantified within the linear range using standard curves of representative standards. The response factors were determined from freshly prepared solutions in the concentration ranges 2–250 mg/L.

### Statistical analysis

Results for antimicrobial tests were reported as means ± standard deviation. Two-tailed, unpaired Student’s t-test (Microsoft Excel 2007, Microsoft Corp., Santa Rosa, CA, USA) was used to calculate the statistical significance of the differences between CFUs from plated bacteria-broth/PBS controls and plated bacteria-honey mixtures. Significance was defined as a value of p of <0.01.

## Results

### Antimicrobial activity of honey

**Figure 1:**
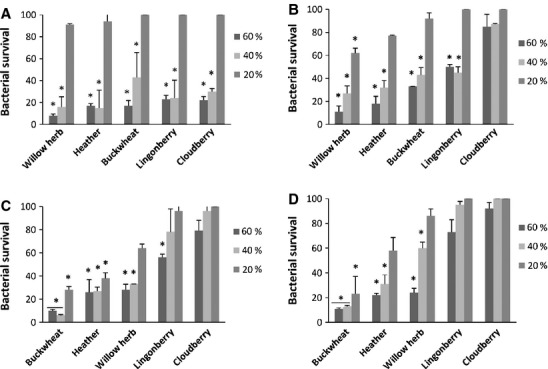
Antimicrobial activity of buckwheat, cloudberry, heather, lingonberry, and willow herb honeys (60%, 40%, and 20%) concentrations (w/v) against *Streptococcus pneumoniae* (A), *Streptococcus pyogenes* (B), *Staphylococcus aureus* (C), and methicillin-resistant *S. aureus* (D). The values represent the mean and standard deviation calculated from two individual experiments. Bacterial survival was compared to control. Standard deviation, n = 6, *p < 0.01 against the bacterial control.

Antimicrobial activities of five Finnish monofloral honeys (buckwheat honey, cloudberry honey, heather honey, lingonberry honey, and willow herb honey) were studied against human pathogens *S. pneumoniae*, *S. pyogenes*, *S. aureus* and MRSA. In this study we found that all the tested bacteria: *S. pneumoniae*, *S. pyogenes*, *S.aureus* and MRSA were susceptible (p < 0.01) to the tested willow herb, heather and buckwheat honeys (Fig. 1). *S. pneumoniae*, *S. pyogenes*, and *S. aureus,* but not MRSA were also susceptible to the lingonberry honey. The studied cloudberry honey was active only against *S. pneumoniae*. The detected antimicrobial effect was mostly growth inhibiting (results not shown). In the presence of ampicillin (80 µg/mL) there was no bacterial growth, except in MRSA cultures (8% compared to broth/PBS control).

*S. pneumoniae* was significantly (p < 0.01) sensitive to all the tested honeys compared to the control at the honey concentrations of 60% and 40% (Fig. 1A). Best activities were obtained with the willow herb honey. Pneumococcal survival was 8% when 60% concentration of the willow herb honey was used and 16% with 40% willow herb honey. For the heather honey the respective survivals were 17% and 15%, for the buckwheat honey the respective survivals were 17% and 43%, for the lingonberry honey the survivals were 23% and 24%, respectively, and for the 60% and 40% cloudberry honey the respective survivals were 22% and 30%.

The growth of *S. pyogenes* was inhibited significantly (p < 0.01) by all the studied concentrations (60%, 40%, and 20%) of the willow herb honey, when bacterial survival was 11%, 27%, and 62%, respectively (Fig. 1B). Also 60% and 40% concentrations of the heather, buckwheat, and lingonberry honeys had antimicrobial effect (p < 0.01). The corresponding bacterial survivals for the heather honey were 18% and 32%, respectively, for the buckwheat honey 33% and 43%, respectively, and for the lingonberry honey the bacterial survivals were 50% and 45%, respectively, The studied cloudberry honey had no antimicrobial effect against *S. pyogenes*.

The growth of *S. aureus* was inhibited by the buckwheat and the heather honeys in all the tested concentrations of 60%, 40%, and 20% (p < 0.01) (Fig. 1C). The bacterial survival was 10% for the 60% buckwheat honey, 6% survival for the 40% buckwheat honey and 28% survival for the 20% buckwheat honey. In the presence of the heather honey the survival of *S. aureus* was 26%, 27%, and 38% vs the 60%, 40%, and 20% heather honeys, respectively. The willow herb honey of 60% and 40% had significant antimicrobial activity as well (p < 0.01) with the respective bacterial survivals of 28% and 33%. The 60% lingonberry honey had significant antimicrobial activity (p < 0.01) with the bacterial survival of 56%. The tested cloudberry honey was ineffective against *S. aureus*.

There was no significant difference between antibiotic-resistant and antibiotic-sensitive *S. aureus* strains as regards sensitivity to studied honeys which supports the previous findings 29–30. Against MRSA the studied buckwheat honey was the most active and all the tested concentrations had significant effect against the growth of MRSA. For 60% buckwheat honey MRSA survival was 11%, for 40% buckwheat honey MRSA survival was 13% and for 20% buckwheat honey MRSA survival was 23% (Fig. 1D). The heather and the willow herb honeys had significant antimicrobial activity at 60% concentration and the bacterial survival was 22% and 24%, respectively. Furthermore, at 40% concentrations of the heather and the willow herb honeys significant antimicrobial activity was also found with 31% and 60% bacterial survival, respectively. Neither the tested cloudberry honey nor the lingonberry honey had antimicrobial activity against MRSA.

### Phenolic compounds in honey

The honey samples contained only minor amounts of phenolic acids, benzoic acid, and flavonoids. The heather honey contained the highest amount of phenolic compounds, 8.68 mg/100 g of honey of which 7.07 mg was benzoic acid. The lingonberry honey contained phenolic compounds 4.21 mg/100 g, mostly benzoic acids (1.48 mg) and hydroxybenzoic acid (2.46 mg). The buckwheat honey contained phenolic compounds 2.91 mg/100 g, most of them were benzoic and hydroxybenzoic acids, 1.07 and 1.1 mg, respectively, and 0.67 mg of hydroxycinnamic acids. The cloudberry honey contained 1.13 mg/100 g phenolic compounds, which was hydroxybenzoic and hydroxycinnamic acids. The willow herb honey contained only 0.65 mg of phenolic compounds/100 g of honey in total (Table 1).

**Table 1:** Contents of phenolic compounds in honey samples with HPLC-DAD method

	Contents of phenolic compounds (mg/100 g of honey)					
Finnish monofloral honey	Buckwheat	Cloudberry	Heather	Lingonberry	Willow herb
Total	2.91	1.13	8.68	4.21	0.65
Benzoic acid	1.07	ND	7.07	1.48	0
Hydroxybenzoic acids	1.1	0.8	0.98	2.46	0.4
Hydroxycinnamic acids	0.67	0.33	0.29	0.22	0.12
Flavonoids^1^	0.07	ND	0.34	0.05	0.13

1 Compounds with a flavonoid kind of spectrum are quantified as quercetin glucoside equivalents.

## Discussion

The studied bacteria: *S. pneumoniae*, *S. pyogenes*, *S. aureus* and MRSA are all important pathogens causing various infections and their antibiotic resistance is a significant problem worldwide 1,4. Here we describe significant antimicrobial activity of Finnish honey products, buckwheat honey (*F. esculentum*), cloudberry honey (*R. chamaemorus*), heather honey (*C. vulgaris*), lingonberry honey (*V. vitis-idaea*), and willow herb honey (*E. angustifolium*), against these bacterial pathogens for the first time.

When considering topical therapy of honey in wound healing 14 or eradicating biofilm forming rhinosinusitis 32 high concentrations of honey are used. In licensed wound products, honey concentration is usually over 80% 14. According to literature, active honey concentrations can be as low as 3.1% 33. In our study, we used honey concentrations of 60%, 40%, and 20% and the highest antimicrobial activity was achieved, when 60% and 40% honeys were used, but significant antimicrobial effect with 20% honey was also detected. Against *S. pyogenes,* 20% willow herb honey was significantly active with bacterial survival of 62%, against *S. aureus* 20% buckwheat and heather honeys were significantly effective (bacterial survival 28% and 38%, respectively) and against MRSA 20% buckwheat honey had significant antimicrobial activity with bacterial survival of 23%.

The traditional use of honey in hot drinks could possibly have a role as a topical treatment against nasopharynx colonizing pathogens, *S. pneumoniae*, *S. pyogenes,* and *S. aureus* associated with respiratory infections 3–34. Against *S. pneumoniae*, a short heat treatment (68 °C, 15 min) of 60% willow herb, heather, and buckwheat honeys, did not affect their antimicrobial activity (results not shown). Neither did heating reduce the bioactivity of the willow herb and heather honeys against *S. pyogenes* nor the bioactivity of the buckwheat honey against *S. aureus* or MRSA (results not shown). High osmolarity, low pH, and hydrogen peroxide are the main antimicrobial factors in honeys 15,16. Because heating reduced some, but not all the bioactivity of the tested honeys, it gives a clue that hydrogen peroxide may have a role, but there are also other contributing antimicrobial factors in these honeys.

In our study, we found that the 20% concentrations of willow herb, heather, and buckwheat honeys had significant antimicrobial activity at neutral pH. This refers to other contributing antimicrobial factors than high osmolarity or pH in buckwheat, heather, and willow herb honeys. Cloudberry honey was not active against *S. pyogenes*, *S. aureus* or MRSA at the 60% concentration. This further supports the finding that the antimicrobial activity against these bacteria was not due to high osmolarity of the studied honeys.

Phenolic content varies between different honeys, and phenolic compounds may contribute to antimicrobial activities in honeys 18. In the studied Finnish honeys only very small amounts of phenolic acids, benzoic acid, and flavonoids were present. The highest concentration of phenolic compounds was detected in heather honey, almost 9 mg/100 g honey, which composed mostly of benzoic acid. Heather honey was active against all the studied bacteria, and thus benzoic acid may possibly have an additional role as an antimicrobial agent in the studied honeys. However, for antimicrobial activity of berry phenolic compounds, at least 100 times higher concentrations were needed for antimicrobial activity against *S. pneumoniae*, when the same assay methodology was used 24. In the studied honeys, benzoic, hydroxybenzoic, and hydroxycinnamic acids were found in free forms and flavonoids were detected as aglycones and glycosides. In berries benzoic, hydroxybenzoic sand hydroxycinnamic acids occur predominantly as sugar esters and glycosides 27–28. This difference may have an effect on the antimicrobial activity of phenolic compounds in honeys. The total concentration of the phenolic compounds in the two other active honeys against all the studied bacteria, willow herb, and buckwheat honeys, were very low, 0.65 and 2.91 mg/100 g of honey, respectively, and thus phenolic compounds do not explain their antimicrobial activity. Characterization of the unknown active components in Finnish honeys remains to be carried out in future.

Against *S. pyogenes*, *S. aureus* and MRSA several honeys with different floral backgrounds have been reported to possess antimicrobial activity 9–30. Parallel to our results (Fig. 1C) against *S. aureus*, heather, and buckwheat honeys from different geographical origins have been previously reported to be active 8–22. There are honeys that do not possess any antimicrobial activity against certain bacteria. Here we show that the studied cloudberry honey was inactive against *S. pyogenes*, *S. aureus,* and MRSA strains, but was antimicrobial against *S. pneumoniae*. This demonstrates higher sensitivity of *S. pneumoniae* to varying levels of antibacterial compounds present in honey samples. Variations in antimicrobial activity and active components between different honeys may result from different geographical locations and floral sources, as well as differences in harvesting, processing, storage conditions, and bee-related factors 23–33.

Honey is a natural, nontoxic, and inexpensive product for the need of novel therapies against bacterial infections. The clinical use of honey has an enormous potential, especially in the fight against antibiotic-resistant strains. Here we show antimicrobial activity and anti-infective potential of Finnish monofloral willow herb, heather, buckwheat, lingonberry, and cloudberry honeys for the first time against important human pathogen *S. pneumoniae*. In this study we also show that against *S. pyogenes*, *S. aureus*, and MRSA bacteria the tested willow herb, heather, and buckwheat honeys had significant antimicrobial activity. Future studies are needed both to reveal the active components and to clinically prove the efficacy of these Northern honeys against the tested pathogens.

We thank Finnish Beekeepers Association for providing the honey samples, and Jukka Hytönen and Sauli Haataja for bacterial strains. We also thank Sari Ukkonen and Eeva-Liisa Palkispää for skilful technical assistance. This study was funded by the European Regional Development Fund and the Finnish Funding Agency for Technology and Innovation (TEKES) (number 70096/08) together with Fazer Makeiset Oy (Vantaa, Finland), Kiantama Oy (Suomussalmi, Finland), VIP-Juicemaker Oy (Kuopio, Finland), and the Finnish Beekeepers Association.
